# Hypertrophic cardiomyopathy in the trauma patient: A case report and review of the literature

**DOI:** 10.1016/j.tcr.2025.101204

**Published:** 2025-05-26

**Authors:** Andrew Hendrix, Thomas Crafton, Logan Carlyle, Jacob Hessey

**Affiliations:** a University of South Carolina School of Medicine, 6311 Garners Ferry Road, Columbia, SC 29209, USA; b Prisma Health Midlands, Department of General Surgery, 2 Medical Park Rd Ste 300, Columbia, SC 29203, USA; c Prisma Health Midlands, Department of Internal Medicine, 2 Medical Park Rd Ste 203, Columbia, SC 29203, USA; d Prisma Health Midlands, Department of Trauma Surgery, 2 Medical Park Rd Ste 300, Columbia, SC 29203, USA

**Keywords:** Hypertrophic cardiomyopathy, Cardiogenic shock, Trauma surgery, Hemodynamics, Cardiology

## Abstract

**Introduction:**

Hypertrophic cardiomyopathy (HCM), a prevalent genetic cardiac condition characterized by myocardial thickening, poses unique challenges in trauma care. Sparse evidence seems to agree that HCM patients have worse outcomes following non-cardiac surgeries, particularly emergent procedures. However, despite a prevalence of 1 in 500 in the US population, the intersection of HCM and Advanced Trauma Life Support (ATLS) remains undiscussed in current literature.

**Case presentation:**

A 54-year-old female with unknown past medical history presented as a level 2 trauma alert following a motor vehicle collision. Due to persistent hypotension and transient bradycardia in the trauma bay a further cardiologic evaluation was performed and found severe hypertrophic obstructive cardiomyopathy (HOCM).

**Clinical discussion:**

We report a case of a polytrauma patient found to have severe HOCM as well as provide a review of the literature including pathophysiological considerations in the management of the trauma patient with HCM.

**Conclusions:**

Caring for the trauma patient with HCM requires a multidisciplinary strategy that integrates advanced cardiac imaging and cautious hemodynamic management, underlining the necessity for heightened awareness.

## Highlights

• It is important to recognize HCM as a contributor to shock in the trauma patient.
• HCM patients have worse outcomes following emergent non-cardiac surgeries.
• Focus on lengthening diastolic filling time and maintaining a hypervolemic state
• Phenylephrine is the preferred vasopressor.

## Introduction

Hypertrophic cardiomyopathy (HCM) is an inherited cardiac anomaly distinguished by pathological thickening of the myocardium, occurring asymmetrically and without apparent causative factors or medical history [1]. Affecting approximately 1 in 500 individuals in the US [2,3], this condition is further categorized as obstructive or non-obstructive, with hypertrophic obstructive cardiomyopathy (HOCM) often characterized by obstruction of the left ventricular outflow tract (LVOT), frequently due to systolic anterior movement (SAM) of the anterior mitral valve leaflet as seen on echocardiography [1,4]. An LVOT gradient at rest measuring ≥30 mmHg may indicate potential obstruction [1]. The pathogenesis of HCM involves a monogenic disease stemming from mutations in one of at least 13 currently identified sarcomeric genes, typically inherited in an autosomal-dominant manner [2,5]. These mutations lead to disorganized collagen deposition as well as hypertrophy of myocytes resulting in irregular thickening of the interventricular septum [2,5]. Contrary to earlier assumptions associating HCM primarily with young athletes, recent data from the Hypertrophic Cardiomyopathy Registry (HCMR) reveals an average onset age of 49 years, with a higher incidence among males (71 %) [6]. Notably, a significant proportion of patients remain asymptomatic until late stages of the disease resulting in presentations including heart failure symptoms, chest pain, arrhythmias, syncope, and, in rare instances, acute hemodynamic collapse [7]. Diagnostic approaches typically involve echocardiography or cardiac magnetic resonance imaging (MRI), with electrocardiography (EKG) serving to establish baseline electrical changes [1,8]. EKG findings typically reflect left ventricular hypertrophy (LVH), left atrial enlargement, as well as ST depressions and T wave inversions [2,9]. Diagnostic criteria include a left ventricular wall thickness ≥ 15 mm at any single point or ≥ 13 mm in patients with a positive family history of HCM [1]. The management of HCM involves utilizing beta blockers and non-dihydropyridine calcium-channel blockers, to help alleviate LVOT obstruction by lengthening diastolic filling time to the hypertrophied left ventricle. [10] Complications associated with HCM pose significant risks, particularly in terms of adverse cardiac events such as arrhythmias, including ventricular fibrillation and sustained ventricular tachycardia. [1] Cohort studies suggest an annual mortality estimate of 1 % [1,11,12], with approximately 20 % of patients also developing atrial fibrillation over time [1].

Management of hypertrophic cardiomyopathy becomes especially nuanced in the perioperative settings where meticulous attention to cardiac function and hemodynamic stability is paramount. If the patient has a known diagnosis of HCM prior to surgery, pre-procedure evaluation necessitates a comprehensive echocardiogram to assess the severity of LVOT gradient, identify SAM, and ascertain the degree of LVH [13]. During induction of anesthesia, care should be taken to attenuate the sympathetic response while ensuring optimal intubating conditions. Preemptive administration of intravenous metoprolol or esmolol helps mitigate tachycardia during laryngoscopy [14]. Intraoperatively, maintenance of anesthesia favors the use of volatile agents for their myocardial depressant properties [14]. Additionally, invasive arterial access is recommended for continuous hemodynamic monitoring [13,14]. Appropriate preload is an important factor in preventing LVOT obstruction and should be maintained at levels higher than normal with avoidance of vasodilators, diuretics, and hypotonic fluids [13,14]. Careful control of heart rate and contractility may be achieved through beta blockers or non-dihydropyridine calcium-channel blockers, with a goal heart rate of 60–80 beats per minute [13,14]. Afterload maintenance is also a crucial part of intraoperative management, with phenylephrine being the preferred vasopressor due to its ability to increase systemic vascular resistance without augmenting contractility [13,14]. Of note, transesophageal echocardiography may be warranted for patients with significant hemodynamic shifts or suspected SAM [15] and management of acute atrial fibrillation typically involves direct current cardioversion for immediate reversal [11]. Postoperative care focuses on preventing sympathetic-induced exacerbation of LVOT obstruction. Pain relief should be diligently managed, and if tachycardia ensues, intravenous metoprolol can be administered incrementally [14]. Additionally, beta-blockade should not be withheld postoperatively, especially in patients with borderline systolic blood pressures, as it helps alleviate tachycardia-induced exacerbation of obstruction which may in fact be the driving force behind the hypotension [14]. Bedside echocardiography is a helpful tool to assess LVOT obstruction and may clarify the need for beta blockers in this context. Overall, a multidisciplinary approach integrating cardiovascular expertise is essential for optimizing outcomes in patients with HCM undergoing surgical interventions.

## Case presentation

**Fig. 1:**
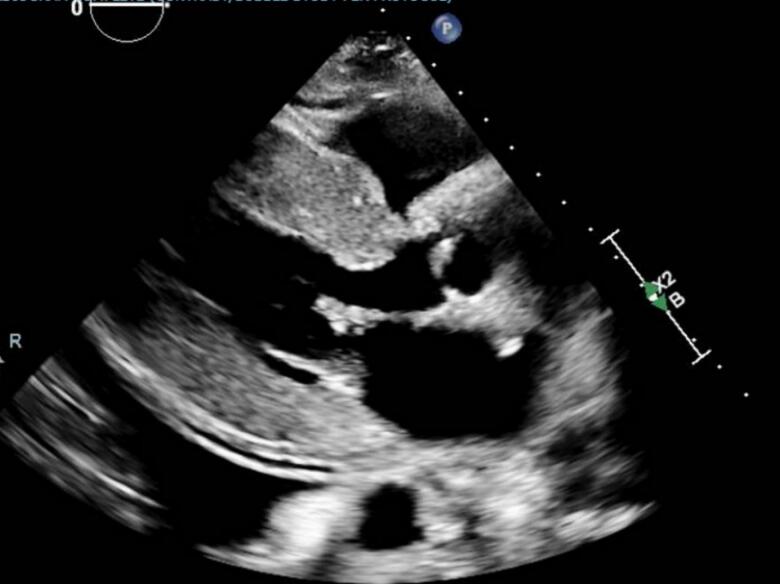
Echocardiography demonstrating left ventricular hypertrophy.

A 54-year-old female with unknown past medical history presented as a level 2 trauma alert following a motor vehicle collision. Upon arrival in the trauma bay, the patient was evaluated following ATLS guidelines. The patient was intubated for airway protection in the setting of a Glasgow Coma Scale (GCS) <8 and taken to the CT scanner for imaging which revealed a grade 1 liver laceration with mild surrounding hematoma, a type II C2 dens fracture with posterior displacement, and several fractured ribs. During ED evaluation the patient had multiple transient episodes of bradycardia down to 30 beats per minute with concurrent hypotension. Initially it was unclear whether the shock was cardiogenic, associated with the bradycardia, or hemorrhagic due to the liver laceration. The patient was resuscitated with two units of packed red blood cells and cardiology was consulted to further characterize the type of shock. EKG and troponins were unremarkable but echocardiography showed severe left ventricular hypertrophy with severe left ventricular outflow tract (LVOT) obstruction with a LVOT gradient over 90 mmHg (Fig. 1). Severe mitral regurgitation with systolic anterior motion of the mitral valve annulus was also noted. Patient was started on diltiazem 30 mg every 6 h via NG tube to prolong diastolic filling and maximize preload. Calcium channel blocker were chosen as opposed to beta-blocker to avoid recurrence of the bradycardic episodes, although bradycardia was permissible in the event she remained hemodynamically stable otherwise. Regarding perioperative management, cardiology recommended maintaining the patient in a mildly hypervolemic state to maintain preload. The patient required surgical intervention for her C2 fracture with the neurosurgery service where she underwent odontoid screw placement. Prior to the procedure, the patient underwent general anesthesia with propofol. Intraoperatively, damage to the left vertebral artery was noted requiring angiogram and coiling. This complicated the patient's hemodynamic management even further as now strict systolic blood pressure (SBP) goals (100-140 mmHg) were placed to ensure adequate brain perfusion. SBP goals were maintained with both phenylephrine and nicardipine, while lactated ringer was continued at 100 ml/h. Post-operatively, the patient was managed in the STICU by the trauma service due to her number of severe traumatic injuries. On hospital day 6 the patient presented with worsening respiratory distress. CTA confirmed a pulmonary embolism and bilateral pleural effusions were present. Ultrasound of bilateral lower extremities were negative for deep vein thromboses. Patient was started on heparin and bilateral thoracostomy tubes were placed. Due to continuous bleeding from tube sites the decision was made to discontinue heparin and place an IVC filter. On hospital day 14, management of the patient's hemodynamic status was further complicated by the development of septic shock secondary to pseudomonas UTI. The patient was started on cefepime and cardiology was again consulted to assist with vasopressor management and the patient was started on phenylephrine and vasopressin as opposed to norepinephrine. Due to the fact the patient's circulatory status was complicated by lack of forward flow in the setting of HOCM and mitral regurgitation, aggressive fluid resuscitation was performed to maintain preload despite an elevated BNP. The patient required tracheostomy and PEG placement on hospital day 21 and was ultimately able to be discharged to an acute rehabilitation facility.

## Discussion

We report a case of a polytrauma patient found to have severe HOCM. The obstruction of her LVOT had a significant impact in the management of her hemodynamic status throughout the hospital admission. Careful consideration was made in medication selection and fluid management both in the perioperative period and when the patient developed septic shock. This case serves as a strong example of maintaining a heightened suspicion for HOCM in the hemodynamically unstable trauma patient as well as the importance of a multidisciplinary approach in management.

Shock is a common presentation in the trauma bay and while resuscitation and stabilization are a central part of ATLS, so too is the investigation into the etiology for hemodynamic instability. The most common reason for shock in the trauma setting is hypovolemia secondary to blood loss [16,17]. This may be obvious if bleeding is external or require imaging modalities such as FAST exam or CT if bleeding is internal. A second, less common etiology of shock is known as cardiogenic in which the heart fails to adequately circulate blood to the body. This is often seen in the setting of cardiac tamponade or severe heart failure exacerbations. In our case, both hypovolemic shock secondary to the liver laceration and cardiogenic shock secondary to HOCM were at play in a synergistic manner. As the patient lost more blood, the LVOT obstruction worsened further compromising her circulation. While fluid resuscitation was an essential part in her stabilization, identifying LVOT obstruction during the FAST exam was ultimately the key factor in management of the patient in the trauma bay, perioperative setting, and STICU admission when she developed sepsis.

Despite an estimated prevalence of 1 per 500 persons in the US population [2], HCM has not received the same attention in the literature when it comes to management considerations in the trauma patient. The limited evidence that is present, shows a clear relationship between HCM patients and worse outcomes when undergoing noncardiac surgical procedures [[18], [19], [20], [21], [22]], particularly emergent procedures [20]. This evidence supports maintaining a heightened suspicion for cardiogenic shock secondary to HOCM in the hypovolemic shock patient who is not responding properly to fluid resuscitation. It is in this patient that the pericardial view during the FAST exam can prove vital in navigating stabilization efforts.

## Conclusion

This report critically reviews pathophysiological considerations of HCM in the trauma patient and provides a case highlighting the complex management of a trauma patient with HOCM. Ultimately, caring for this patient population requires a multidisciplinary strategy that integrates advanced cardiac imaging and cautious hemodynamic management, underlining the necessity for heightened awareness and specific competencies among trauma care providers.

## CRediT authorship contribution statement

**Andrew Hendrix:** Writing – original draft, Visualization, Methodology, Investigation. **Thomas Crafton:** Writing – review & editing, Visualization, Conceptualization. **Logan Carlyle:** Writing – original draft, Visualization, Investigation. **Jacob Hessey:** Writing – review & editing, Supervision, Project administration, Methodology, Conceptualization.

## Consent

Informed consent has been obtained from all individuals included in this study.

## Funding

This research did not receive any specific grant from funding agencies in the public, commercial, or not-for-profit sectors.

## Declaration of competing interest

The authors declare that they have no known competing financial interests or personal relationships that could have appeared to influence the work reported in this paper.
